# Identifying key factors in building fires: A novel approach fusing K-shell entropy gravity

**DOI:** 10.1371/journal.pone.0350804

**Published:** 2026-06-05

**Authors:** Yongping Yu, Ning Wang, Shibo Cui, Enhui Zhao

**Affiliations:** School of Economics and Management, Dalian University of Technology, Liaoning, China; The University of Alabama in Huntsville, UNITED STATES OF AMERICA

## Abstract

Building fire key factors are the fundamental control variables that govern both the initiation of fires and dynamics of propagation. The accurate identification of key factors in building fires is crucial for enhancing the effectiveness of fire prevention strategies. To improve the accuracy of key factor identification in building fires, a novel K-shell Entropy Gravity (KEG) algorithm that integrates multiple topological metrics is proposed in this study. First, a complex network is constructed to characterize the relationships among accident factors, where nodes represent influencing factors and edges denote their co-occurrence in fire incidents. Subsequently, considering the positional importance and core connectivity of nodes, the information influence and irreplaceability of nodes, as well as the collaborative coupling and nonlinear characteristic among multiple indicators, a composite attribute integrating K-shell value, information entropy difference, and total shortest path length is developed to quantify node importance, thereby capturing both the local coreness and the global influence of nodes within the network. Then, these metrics are incorporated into an established gravity-based model to comprehensively assess the influential scope of each node, and the results are employed to identify the key factors. Finally, the proposed method is compared with baseline methods based on the Susceptible–Infected–Recovered (SIR) model and network robustness evaluation using the California Building Fire Dataset (2012–2024). In addition, a sensitivity analysis is performed to investigate how the removal of key factors affects accident propagation. To further verify the robustness of this method, fire data from Alaska are applied for comparison, and an ablation experiment is designed. The results indicate that the KEG algorithm achieves superior accuracy in identifying critical factors and offers a reliable analytical tool for developing targeted fire prevention and mitigation strategies.

## 1 Introduction

Frequent fire accidents pose severe threats to production, daily life, economic activities, and human safety. Analyzing these accidents is essential to mitigate their impacts. Understanding incident characteristics and contributing factors enables effective control measures to reduce occurrence probability and losses [1].

Building fires, often ignited by combustible materials, spread rapidly, hinder evacuation, complicate rescue, and cause extensive damage. Accurately identifying key factors—such as human negligence, electrical faults, and combustible materials—is critical for loss reduction and safety improvement. These factors strongly influence fire outcomes and interconnect with other variables, making them representative targets for efficient prevention strategies.

Extensive research has been conducted on key factor identification. Kim et al. [2] surveyed Korean fire officials to identify and relate key factors in construction site fires. Kim et al. [3] applied principal component analysis to seasonal fire data. Cai et al. [4] built a logistic regression model to analyze forest fire factors in Zhejiang. Aamodt et al. [5] used relationship networks to identify critical elements in fire spread. Kim et al. [6] assessed fire risk via likelihood, vulnerability, and social impact. Zhang et al. [7] employed finite element analysis to evaluate electrical fire risks. Tang et al. [8] applied bivariate and multivariate regression to UK residential fire data, identifying night fires, occupancy, and human behavior as key. Aydin et al. [9] used fuzzy Bayesian networks for marine fire risks, and Plathner et al. [10] applied logistic regression to examine ignition and environmental influences.

Current methods—logistic regression, statistical analysis, and expert knowledge—can identify key factors but often overlook inter-factor relationships, limiting perspective and accuracy.

Complex networks provide an intuitive abstract model for complex systems. Compared to fault trees [11], Bayesian networks [12], or regression [13], they better represent accident-factor and factor-factor relationships using nodes and edges [14], and are widely used in risk analysis [15–17]. In such networks, factors are nodes, and edges link factors involved in the same accident. Key factors correspond to key nodes [18,19]. Identifying these nodes is crucial. Common metrics like degree [20], closeness [21], and betweenness centrality [22] have limitations: degree only considers direct connections; closeness requires connected networks and ignores path alternatives; betweenness over-relies on shortest paths. These shortcomings reduce node importance assessment reliability. Nandi [23] proposed a new method named IC-SNI. It measures the node influence by aggregating the structural and neighbor information within the two-hop neighborhood of the node (introducing connection strength and effective distance), and verifies its superior performance in identifying influential spreaders on multiple real network datasets. Nandi [24] and his team also proposed a method called Local Closeness Gravity (LCG), which improves the gravity model by combining local closeness centrality within the truncation radius, K-shell index, and connection strength – based information sharing ability to identify important nodes in the network. Li [25] proposed a multi – feature gravitational model (MCGM) that integrates node neighbor quantity, neighbor influence, network location, and path information to address the problem of identifying nodes for influence spread in complex networks. They solved the problem of inconsistent feature dimensions through a normalization strategy and verified the effectiveness of the universal truncation radius. The recognition performance of this model on ten types of real – world networks is superior to that of classical centrality and mainstream gravity – based methods. Yang [26] proposed an improved gravitational centrality method integrating the K – shell index (KSGC) to address the defect that the existing gravitational models do not fully consider the node position information. By introducing an attraction coefficient based on the difference in K – shell values to adjust the gravity between nodes, and combining node degree, distance, and position information, experiments on real and synthetic networks show that its performance in identifying influential nodes is superior to traditional centrality and mainstream gravity – based and K – shell – based methods. While these methods have made significant progress in identifying influential nodes by leveraging local structures, multi-feature integration, or gravity models, they still leave room for improvement in terms of comprehensively incorporating global positional information and multi-order neighbor contributions. Therefore, this paper proposes an enhanced approach to further refine node importance evaluation, aiming to achieve higher accuracy and robustness in complex network analysis.

Accurately identifying key factors is essential for controlling building fires. Based on existing research, this study proposes a K-shell Entropy Gravity (KEG) algorithm integrating multiple models to improve accuracy. A complex network is built from NFIRS data on California (CA) building fires (2012–2024). For each node, the K-shell value, entropy, and total shortest path length are computed. Node influence is then evaluated via a gravity model to identify key factors. The KEG algorithm’s effectiveness is validated using an SIR model and robustness tests, with sensitivity analysis further confirming result accuracy. Apply the fire data in Alaska for comparison and design ablation experiments. The approach enables precise identification of key factors.

The paper is structured as follows: Chapter 2 describes the data and its preprocessing. Chapter 3 details the methodology. Chapter 4 covers the application and validation of the methods. Chapter 5 discusses the results. Chapter 6 provides the concluding remarks.

## 2. Data description

### 2.1 Study area

**Fig 1:**
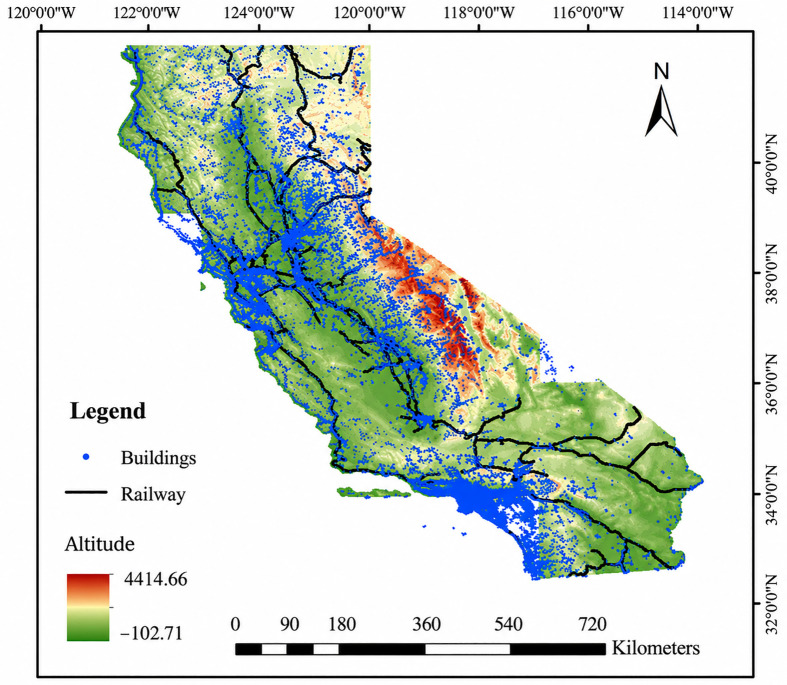
Elevation map of the State of California.

California occupies a position on the southwestern Pacific coast of the United States, with its geographic extent ranging from 32°30′N to 42°00′N in latitude and 114°8′W to 124°24′W in longitude. It covers an area of approximately 424,000 square kilometers, with a coastline extending 2,030 kilometers (Fig 1). The climate is characterized by a distinct Mediterranean pattern, featuring hot, dry summers with scarce precipitation, creating natural conditions conducive to wildfires. In 2023, California’s estimated population was 40.22 million, making it the most populous state in the U.S. In 2022, its gross regional product reached $3.6 trillion, with a per capita GDP of approximately $92,000, ranking as the world’s fifth-largest economy in nominal terms.

California exhibits a high level of urbanization, with dense and diverse building types [27]. However, older structures often lack modern fire suppression systems, posing significant risks. On July 25, 2025, a major fire broke out at the University Garden Shopping Center [28], causing enduring negative impacts on residents’ livelihoods, economic development, and the ecological environment. The diversity of building types and heterogeneity of the population underscore the importance of fire prevention and emergency management. Therefore, it is imperative to investigate and identify the key drivers of fire incidents at their root to mitigate risks effectively, which is helpful for implementing interventions to reduce the occurrence of fires, and minimizing both the probability of fires and associated human and economic losses.

### 2.2 Data processing

The data preprocessing methods in the article are not standard public methods and are processed to meet the subsequent analysis requirements. The processing process is as follows: this study utilized fire incident data obtained from the National Fire Incident Reporting System (NFIRS), a comprehensive national database encompassing fire incidents reported across the United States [29]. The dataset spanned the period from January 2012 to December 2024. Three primary modules were extracted for analysis: the Basic Incident Module, the Fire Module and the Address Module.

To ensure the integrity and reliability of the subsequent analysis, a rigorous data cleaning and quality control protocol was implemented. Initially, records from the state of California were identified and isolated using the ‘STATE’ field. Subsequently, records with critical missing values in key identifiers—namely ‘FDID’ (Fire Department Identifier), ‘INC_DATE’ (Incident Date), ‘INC_NO’ (Incident Number), and ‘EXP_NO’ (Exposure Number)—were excluded. Further filtering was applied to retain only incidents where the INC_TYPE (Incident Type) code specifically indicated a fire incident, thereby removing non-fire-related emergencies. Inconsistencies, such as invalid date entries or illogical values (e.g., incident dates set in the future), were identified and rectified or removed. Duplicate entries, detected based on the unique combination of ‘FDID,’ ‘INC_DATE,’ ‘INC_NO,’ and ‘EXP_NO,’ were also eliminated.

Following the cleaning process, the three modules were meticulously integrated to generate a more complete dataset using the aforementioned variables as a composite key. This multi-step preprocessing yielded a consolidated, high-quality dataset specific to fire incidents in California. The final dataset encompasses 89 distinct factors related to fire causation, spread, and outcomes. These factors were systematically classified into 16 coherent categories—such as Misuse of Material or Product, Mechanical Failure, and Equipment Malfunction—to facilitate analysis. A complete description of all variables and the classification scheme is available in the supporting information (S1 File).

## 3 Method

### 3.1 Methodology framework

**Fig 2:**
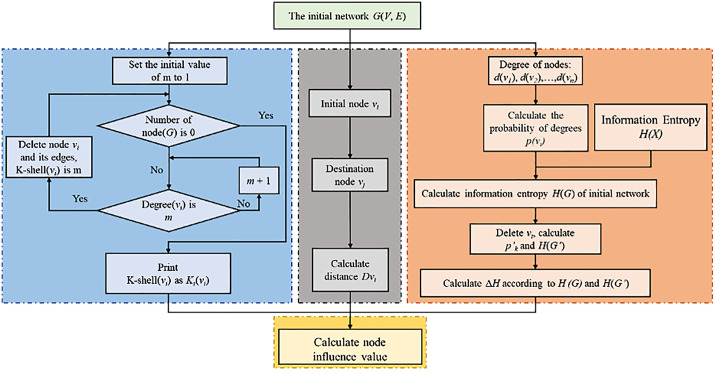
The proposed KEG algorithm.

Fig 2 illustrates the flowchart of the proposed KEG model, which is constructed around three functionally independent modules. The blue dashed module executes the K-shell decomposition algorithm, mapping nodes to their corresponding hierarchical positions within the core-periphery topology of the network. The output of this module characterizes the depth of a node’s embedding in the structural backbone of the network—the higher the K-shell value of a node, the greater its significance. The orange dashed module focuses on information-theoretic attribute extraction by computing the entropy difference (i.e., differential entropy) of the network system under two states: with the target node present and removed. A larger entropy difference indicates that the node serves as a critical point for information flow, implying higher importance. The gray dashed module quantifies the spatial accessibility of a node within the network by calculating the cumulative shortest-path distance from the target node to all other nodes. Nodes with smaller cumulative distances are geographically closer to the overall network structure, enabling more efficient interaction with other nodes and thus possessing higher significance.

The most notable innovation of the KEG model lies in its paradigm for nonlinearly integrating the three aforementioned attributes, which is directly inspired by the theoretical logic of Newton's law of universal gravitation. Within this framework, the “coreness” output by the blue module and the “information-theoretic impact” output by the orange module are synergistically integrated to form a “comprehensive nodal influence” term. These two quantities are analogous to the “product of the masses” in gravitational theory, representing the inherent “attractiveness” of a node. Conversely, the “squared cumulative distance” output by the gray module serves as a “positional resistance” term, corresponding to the “square of the distance” in gravitational theory, which modulates the strength of interaction between nodes. By defining the node importance index as the ratio of “comprehensive influence” to “positional resistance,” the KEG model establishes a theoretically self-consistent quantitative framework that naturally captures the interplay among structural, informational, and positional attributes.

### 3.2 Accident-factors network construction

Based on the complex network theory, a linkage mechanism relying on real incident data is established, and a deep risk analysis model grounded in the co-occurrence relationships of risk factors and the system’s topological structure is constructed. Each specific risk factor identified in fire investigation reports (“aging electrical wiring”, “unauthorized hot work”) is modeled as a node in the network.

If two factors co-occur in the same building fire accident, an undirected weighted edge is established between their corresponding nodes. The weight of the edge represents the frequency of co-occurrence of the two factors across all accidents, forming a data-supported connection. The network is denoted as *G* = (*V*, *E*, *W*), where *V* represents the set of nodes, *E* the set of edges, and *W* the set of edge weights. The adjacency matrix *A* = (*a*_*ij*_)_*N*×*N*_ of graph *G* is an *N* – order square matrix, and the element *a*_*ij*_ in the *i*th row and *j*th column is defined as follows:


$$\begin{array}{c} a_{ij}=\left\{ \begin{array}{c} @l@w_{ij},\;\;if\text{ there is an edge with weight }w_{ij}\text{ between }i\text{ and }j\\ 0,\;\;\;\;if\text{ there is no edge between }i\text{ and }j \end{array}\right. \end{array}$$ (1)

where *w*_*ij*_ representing the weight of that edge.

### 3.3 Calculation of factors eigenvalues

#### 3.3.1 Calculate the hierarchical values of factors.

By iteratively peeling the complex network, the hierarchical levels of factor nodes are determined. The closer a node is to the core layer, the more critical the corresponding factor is considered within the network. The degree *d*_*i*_ of a node *v*_*i*_ is defined as the number of edges connected to it, where edge weights are treated as multiple edges (i.e., weighted edges are considered as repeated links), and the degree calculation includes these multiple edges. The set of degrees for all nodes is denoted as *D* = {*d*_*1*_, *d*_*2*_, …, *d*_*n*_}. Initialize the core value with *k*_*s*_ = 1, collect the set of factor nodes *S*_*ks*_ in the current subgraph whose degrees are equal to *k*_*s*_, and remove these nodes and their associated edges from the network. After each removal, the degrees of the remaining factor nodes are recalculated within the current subgraph only. This process is iteratively repeated until there are no nodes with degree equal to *k*_*s*_ in the subgraph or until the subgraph becomes empty. When a factor node is removed, its core value is recorded as the current iteration’s *k*_*s*_. Upon termination of the algorithm, all nodes are assigned to different hierarchical levels. The *k*_*s*_ -core subgraph consists of internal nodes with degrees of at least *k*_*s*_ + 1 and their associated edges, forming a nested core structure.

#### 3.3.2 Calculate the information entropy of factors.

Quantifying the importance of nodes using information entropy enables the capture of non-local influences within the network. First, the information entropy of the original network is calculated. Then, the entropy is recalculated after removing a specific node. A large difference between the two entropy values indicates a significant impact of that node on the network structure, suggesting that the corresponding node is more critical.

For a discrete variable *X* taking values {*x*_*1*_, *x*_*2*_, …, *x*_*n*_} with corresponding probability distribution *P*(*X* = *x*_*i*_)=*p*_*i*_ (where *i* = 1,2,…,*n*, and $\overset{n}{\underset{i=1}{\sum }}pi=1$), the information entropy is defined as:


$$\begin{array}{c} H\left(X\right)=-\overset{n}{\underset{i=1}{\sum }}pilog2pi \end{array}$$ (2)

where *p*_*i*_ is the probability of the event *X* = *x*_*i*_ occurring.

For any node *v*_*i*_ ∈ *V*, the corresponding degree centrality is *d*_*i*_ = |{*v*_*j*_ ∈ *V*|(*v*_*i*_, *v*_*j*_)∈*E*}|, and the normalized probability distribution is:


$$\begin{array}{c} pi=\frac{di}{\overset{\left|V\right|}{\underset{j=1}{\sum }}dj} \end{array}$$ (3)

Then, substituting formula (3) into formula (2), based on the information entropy, the degree of disorder in the connection distribution of a node can be quantified, the formula can be furtherly written as:


$$\begin{array}{c} H\left(G\right)=-\overset{\left|V\right|}{\underset{i=1}{\sum }}\frac{di}{\overset{\left|V\right|}{\underset{j=1}{\sum }}dj}log2\frac{di}{\overset{\left|V\right|}{\underset{j=1}{\sum }}dj} \end{array}$$ (4)

Thus, the information entropy of node *v*_*i*_ in the original network is obtained.

Subsequently, removing the node *v*_*i*_ and its connection edges, the subgraph *G’*=(*V*-{*v*_*i*_}, *E’*) is obtained. Recalculating the normalized probability distribution of node *v*_*k*_ based formula (3), which *v*_*k*_ ∈ *V*-{*v*_*i*_}. The *H*(*G’*) of the new information entropy of the subgraph *G’* is obtained by formula (4).

Finally, the significance of the node *v*_*i*_ is defined by the difference between the two calculated information entropy values, it is:


$$\begin{array}{c} \Delta H=H(G\text{'})\;-\;H(G) \end{array}$$ (5)

Once ΔH> 0, indicating the network’s connection distribution is more uniform after removing factor nodes and the significance of node vi is higher; Otherwise, the network’s connection is more concentrated after the removal of node vi, suggesting that vi may be a peripheral node with relatively low significance. Therefore, the greater value of |ΔH |, the more significant the impact of node vi on the network. This provides a data-driven basis for identifying critical nodes within the complex networks.

#### 3.3.3 Calculate the shortest paths between nodes.

In traditional networks, the distance between nodes appears in the form of edge weights, representing the cost of information propagation between nodes. The larger the distance (weight), the greater the cost of information propagation and the higher the difficulty of propagation.

In this study, the edge weight represents the number of multi-edges between nodes *s* and *v*. That is, the larger the weight *w*_*sv*_, the more multi-edges there are between the two nodes, the closer the connection between the nodes, and the smaller the difficulty of information propagation. Therefore, for adjacent nodes, the larger the weight *w*_*sv*_, the smaller the $\frac{\text{1}}{w_{sv}}$. The $\frac{\text{1}}{w_{sv}}$ is used to represent the propagation distance (cost). For non-adjacent nodes, it is necessary to calculate the distance of multiple weighted edges, that is the $\sum \frac{\text{1}}{w_{sv}}$. The farther the distance between non-adjacent nodes, the larger the $\sum \frac{\text{1}}{w_{sv}}$, and the square term ${\left(\sum \frac{\text{1}}{w_{sv}}\right)}^{\text{2}}$further amplifies this effect. This is in line with the laws of communication: the influence of distance is often non-linear. The current method of calculating the distance between nodes takes into account both the edge weight and the step information between nodes.

Based on this, the Dijkstra algorithm and edge weights are applied to calculate the sum of the shortest paths of nodes, so as to quantify their centrality in the risk system. This process relies on a greedy strategy to iteratively determine the shortest distances, effectively measuring the significance of node in the network. For an undirected weighted graph *G* = (*V*, *E*, *W*), where the edge weights *W* represent the co-occurrence frequency of factors. The proceeds as follows:

***Step 1:*** Set the distance from the source point *s* to itself *dist*[*s*] = 0. For all other vertices v ∈ V, initialize their distances to *dist*[*v*] = ∞ . Create an empty set, *visited*, to record vertices for which the shortest path has been determined. The remaining vertices are maintained in the set *unvisited* = *V*.

***Step 2:*** While *unvisited* is not empty, select the node *u*∈*unvisited* with the minimal distance value, as shown in formula (6).


$$\begin{array}{c} u\;=\;argmin\{dist[v]\;|\;v\in \;unvisited\} \end{array}$$ (6)

Mark *u* as *visited* by moving it from *unvisited* to *visited*, as shown in formula (7).


$$\begin{array}{c} visited\;=\;visited\cup \{u\},\;unvisited\;=\;unvisited\;\backslash \;\{u\} \end{array}$$ (7)

For each neighbor *v* of *u* that remains in *unvisited*, perform a relaxation, if:


$$\begin{array}{c} dist[v]\;>\;dist[u]\;+\;number\;of\;accident\;/\;weight\;(u,\;v) \end{array}$$ (8)

then update:


$$\begin{array}{c} dist[v]\;=\;dist[u]\;+\;number\;of\;accident\;/\;weight\;(u,\;v) \end{array}$$ (9)

***Step 3:*** The algorithm terminates when the *unvisited* set is empty. At termination, the array *dist*[*v*] contains the length of the shortest path from the source *s* to each node *v*.

By summing the shortest path lengths from the source node *s* to all other nodes in the network to calculate the total distance *D*(*s*) is obtained. It reflects the significance of factors for a building fire risk network, which can provide valuable insight for emergency response planning and risk interruption strategies.

### 3.4 Calculate the importance of factor nodes

For the key factor identification, a single metric is often insufficient to comprehensively characterize the importance of a factor node. It is essential to consider multiple dimensions, including the node’s local cohesiveness, its impact on the network’s informational structure, and its global reachability. To achieve a multidimensional quantitative assessment of factor importance, this study proposes the following evaluation model based on the gravity model framework:


$$\begin{array}{c} Im\left(vi\right)=\frac{ks\left(vi\right)⬝\Delta H\left(vi\right)}{D^{\text{2}}\left(vi\right)} \end{array}$$ (10)

where *Im*(*v*_*i*_) is the significance of node *v*_*i*_, *k*_*s*_(*v*_*i*_) is the *k*_*s*_-kernel value of node *v*_*i*_, Δ*H*(*v*_*i*_) is the information entropy, *D*(*v*_*i*_) is the sum of the shortest paths.

The core function of the K-shell decomposition algorithm is to measure the positional importance and core degree of nodes. Its theoretical basis is that in a complex network, the importance of a node depends not only on the number of its direct neighbors (degree centrality), but also on its depth in the network hierarchical structure. The K-shell decomposition algorithm can reveal the hierarchical structure of the network from the outside to the inside by gradually removing nodes with a degree of 1 in the network. In a building fire, some factors may have a small number of connections, but once they fail (such as key fire doors, core circuits), it can lead to the collapse of the entire subsystem. The K-shell value can identify such key factors located in the “core” of the network. The larger the K-value, the more the node is in the core layer of the network, and its failure may trigger a wider range of chain reactions. In addition, the node centrality index only focuses on the local area, while the K- core value provides a global perspective on the position.

The core role of information entropy is to measure the information influence and irreplaceability of nodes. Its theoretical basis is that information entropy is used to measure the degree of disorder or uncertainty of a system. In the node deletion method, by comparing the change in network information entropy before and after deleting a certain node (i.e., Δ*E*), the contribution of this node to network information flow or structural stability can be quantified. In this study, if the removal of a node leads to a drastic change in network information entropy (a large *E* value), it indicates that it plays an indispensable role in maintaining the existing order of the network (information transmission or structural stability). In addition, the K-shell provides a static position, while the information entropy difference provides a dynamic impact assessment of “what if this node is lost”. In a building fire, this can be understood as “how much the uncertainty of the accident development path increases after this factor fails”. The greater the change, the stronger the binding force of this factor on the current system.

The core function of the Dijkstra algorithm is to measure the propagation efficiency and control power of nodes. Its theoretical basis is that the Dijkstra algorithm is a classic algorithm for calculating the single-source shortest path in a weighted graph. The sum of the shortest paths from a node to all other nodes in the network (i.e., the reciprocal of eccentricity or a variant of closeness centrality) reflects the efficiency of this node in the propagation of information, energy, or disasters. In a fire network, if a factor is closely related to all other factors, it can quickly affect or activate other nodes. Conversely, if the *D* value is large, it means that it is in a marginal position and has a weak propagation influence.

The core function of choosing the framework of the universal gravitation formula is to achieve the collaborative coupling and non-linear measurement of multiple indicators. The law of universal gravitation states that the gravitational force between two objects is directly proportional to the product of their masses and inversely proportional to the square of the distance between them. In complex networks, it is often used to simulate the “gravitational force” or the intensity of interaction between nodes. Considering the physical mapping (mass and distance), the product *K* × *E* of the K-value (positional importance) and the information entropy difference (structural importance) of a node is regarded as the “mass” of the node. This means that a node must be in a core position and have a strong information influence to be considered as having a “large mass”. Considering the synergy effect, simple addition (such as *K* + *E* + 1/*D*) is often just a linear superposition. However, using the product form *K* × *E* has non-linear characteristics, if the K value is very large but the *E* value is 0 (or vice versa), the product will be very small, which avoids the one-sidedness of a single indicator. Considering the inverse, square penalty mechanism: dividing by *D*² means that if a node has a large self – attribute (mass) but is not closely related to other nodes in the entire network (a large *D* value), its comprehensive importance will be significantly reduced. This is in line with the logic that “if a key factor is located at the edge of the network, its impact on the whole may be less than that of a slightly less critical factor located at the center of the network”.

In summary, the KEG model actually constructs a “node influence field”: (1) K-shell (K): It defines the “hierarchical position” of a node in the network structure (whether it is a core or peripheral node). (2) Information entropy difference (E): It defines the “information weight” of a node in the system (the degree of chaos of the system when the node is removed). (3) Sum of shortest paths (D): It defines the “propagation radius” of node influence (how much cost it takes to affect the entire network). Combining with formula (10) in the original text, this design takes into account both the properties of the node itself (K, E) and its layout in the global space (D), and couples them through the framework of universal gravitation, which can more accurately identify the key factors of fire.

For the setting of weights, this study did not set weights for *K* and *E*. This is because the algorithm ranks nodes according to their relative importance rather than obtaining absolute values. Any non – zero weighting of *K* or *E* only introduces a global constant multiplier, and the order of ranking remains unchanged. Secondly, the multiplicative coupling of *K* × *E* captures their non – linear synergistic effect – the importance of a node is jointly determined by these two attributes rather than a simple addition. Therefore, this product design avoids the subjectivity and uncertainty inherent in the weight selection required by the additive model while retaining the simplicity of the physical analogy. This enables the algorithm to robustly and objectively identify key nodes.

The proposed new method can quantify the criticality of risk factors. By ranking the factors according to the criticality score, it provides a scientific basis for optimizing the allocation of fire – fighting resources, determining the priorities of prevention and control strategies, and implementing hierarchical fire risk management.

## 4 Application and validation

### 4.1 the result of application

#### 4.1.1 Construction of an accident network.

**Fig 3:**
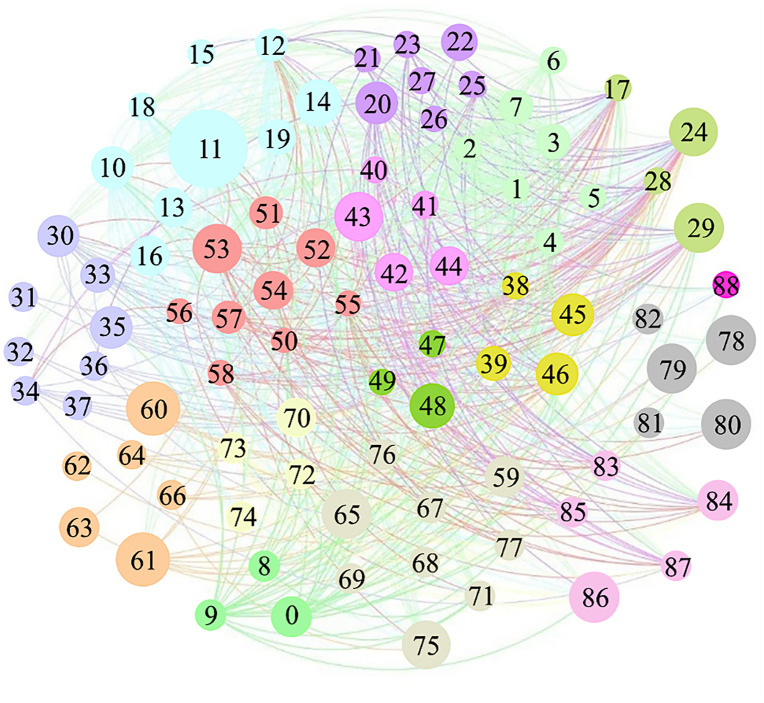
Network model of building fire factors.

To apply the proposed method, a network model is constructed using the data described in the supporting information (S1 File), as shown in Fig 3. The node labels in the model correspond to the codes of individual factors, with larger node areas representing higher degree values. According to the supporting information (S1 File), the factors are categorized into 16 types (e.g., *misuse of material or product*, *mechanical failure or malfunction*, etc.), each represented by a distinct node color in the Fig 3.

The basic attributes of the network are as follow: the number of nodes (𝑁) is 89, the number of edges (𝐸) is 2061, average degree (‹*k*›) is 46.3, assortative parameter (𝑟) is 0.47, propagation theory threshold (*α*_*c*_) is 0.09 and the actual transmission probability (*α*)is 0.15.

#### 4.1.2 Influence ranking of nodes.

To verify whether the constructed network conforms to the basic rules of complex networks, the scale-free and small-world characteristics of the accident network should be examined. The specific verification part can be found in the supporting information (S2 File). After verification, this network conforms to the laws of complex networks, and complex network models and algorithms can be applied.

Using the KEG algorithm, the influence values of the CA network nodes are calculated and sorted. The top ten factors in terms of influence are labeled as follows: factor 10 (Misuse of material or product, other.), which the influence value is 8.63E-03. Factor 6 (Multiple persons involved. Includes gang activity.), which the influence value is 6.83 E-03. Factor 73 (Outside/Open fire for debris or waste disposal.), which the influence value is 6.50 E-03. Factor 12 (Heat source too close to combustibles.), which the influence value is 6.50 E-03. Factor 29 (Electrical arcing.), which the influence value is 6.28 E-03. Factor 43 (Installation deficiency.), which the influence value is 6.28 E-03. Factor 11 (Abandoned or discarded materials or products. Includes discarded cigarettes, cigars, tobacco embers, hot ashes, or other burning matter. Excludes outside fires left unattended.), which the influence value is 6.19 E-03. Factor 2 (Possibly impaired by alcohol or drugs. Includes people who fall asleep or act recklessly or carelessly as a result of drugs or alcohol. Excludes people who simply fall asleep.), which the influence value is 6.09 E-03. Factor 37 (Fluorescent light ballast.), which the influence value is 6.03 E-03. Factor 84 (Heat from direct flame, convection currents spreading from another fire.), which the influence value is 5.84 E-03.

#### 4.1.3 Calculation of time complexity.

To evaluate the computational efficiency of the KEG algorithm for the system, we conduct an analysis of the time complexity of the KEG algorithm. For an undirected weighted network with *N* nodes and *M* edges, its time complexity is calculated as: (1) K-shell: The K- core decomposition algorithm is adopted, with a time complexity of *O*(*M*). (2) Information entropy difference: The information entropy of the network needs to be calculated twice, involving the statistics of the node degree distribution, with a time complexity of *O*(*N*²). (3) Shortest path distance: The Dijkstra algorithm is used to calculate the shortest path from a node to all other nodes. For a weighted graph, the time complexity of the Dijkstra algorithm is *O*(*M* + *N*·log*N*). Since this calculation needs to be performed for N nodes respectively, the total complexity is *O*(*N*·*M* + *N*²·log*N*). (4) Node importance calculation: Calculate *K*·*E*/*D*² based on the framework of the universal gravitation formula, and sort *N* nodes, with a time complexity of *O*(*N*·log*N*). Combining the above steps, the dominant term of the KEG algorithm is *O*(*N*·*M* + *N*²·log*N*). In the case of a dense graph (*M* ~ *N*²), the complexity can reach *O*(*N*³).

The time complexity of the KEG algorithm is relatively high, but the KEG algorithm was initially designed mainly for the specific application scenario of identifying the causal factors of building fires. In such applications, the number of causal factors is usually limited by the complexity boundary of the building system – even considering the most complex building systems, the number of identifiable independent causal factors generally remains within a controllable range (usually on the order of dozens to hundreds). This is mainly because: First, the identification of causal factors of building fires needs to meet the operability of engineering practice. Too fine a division of factors will instead reduce the practical value of the model. Second, the scale of the co – occurrence relationship matrix among factors is limited by the sample size of historical accident data, and the detailed record data of building fire accidents themselves belong to small – to – medium – scale datasets. Therefore, in this application scenario, the computational overhead of the KEG algorithm is completely within an acceptable range.

### 4.2 Validation of effectiveness

The constructed network model is validated to ensure it adheres to the fundamental principles of complex networks, thereby confirming the applicability of various key node identification algorithms. On this basis, a series of comparative experiments are designed to assess the accuracy of the KEG algorithm in identifying critical nodes and to analyze accident sensitivity. More verifications of the KEG method are shown in the supporting information (S3 File).

#### 4.2.1 Verification of node importance by SIR propagation model.

The experiment is designed to validate the significance of the nodes from the perspectives of node transmissibility. The performance of the KEG algorithm is compared with that of the K-shell decomposition algorithm [30], entropy variation [31], gravity model (GM) [32], degree centrality (DC) [20], closeness centrality (CC) [21], betweenness centrality (BC) [22], KEG without K-shell (KEG w/o K-shell), KEG without entropy (KEG w/o entropy), KEG without shortest path (KEG w/o SP), MCGM [25] and KSGC [26] to comprehensively evaluate the effectiveness and accuracy of the proposed model.

The SIR model focuses on evaluating a node’s ability to spread information or viruses within a network. It assumes that each node in the network can be in one of three states: susceptible (*S*), infected (*I*), or recovered (*R*). At each time step *t*, an infected (*I*) node has a probability *α* of infecting its susceptible (*S*) neighbors and a probability *β* of recovering and transitioning to the recovered (*R*) state.


$$\begin{array}{c} \alpha c\;=\text{ ‹}k\text{› }/\;(\text{‹}k^{\text{2}}\text{› }-\text{ ‹}k\text{›}) \end{array}$$ (11)

where ‹*k*› is the average degree of nodes. The SIR propagation experiment is conducted over 50 time steps (*t*).

In this section, the precision function ε is employed to quantify the accuracy of the method proposed in [33], the function is defined as follows:


$$\begin{array}{c} \varepsilon (p)=\text{1}-\frac{Mrank(p)}{Meff(p)} \end{array}$$ (12)

where *p* represents the proportion of evaluated nodes relative to the total network size *N* (p∈[0,1]). *M*_*rank*_(*p*) denotes the average propagation efficiency of the top *pN* nodes ranked by the model according to their influence. *M*_*eff*_(*p*) represents the average propagation efficiency of the top *pN* nodes ranked according to their actual propagation capability. The closer *M*_*rank*_(*p*) is to *M*_*eff*_(*p*), the more accurately the model’s ranking of the top *pN* nodes reflects the actual propagation efficiency of the top *pN* nodes in the network. A smaller value of *ε*(*p*) indicates higher accuracy of the model in identifying key nodes.

**Fig 4:**
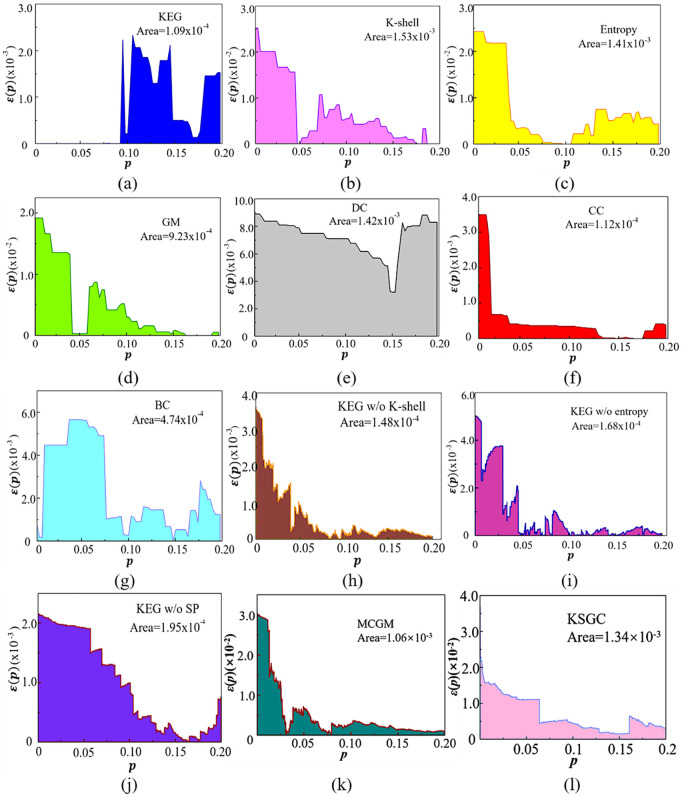
Comparing precision functions for influence ranking methods of nodes in CA accident network.

Fig 4 shows the precision function curves of the spreading capabilities of multiple methods in the CA accident network. The area of the Fig 4 enclosed by the curve and the horizontal axis is marked in each Fig 4. The smaller the area, the closer the ranking of the top *pN* nodes by the model is to their actual spreading efficiency, and the higher the method precision. In the range of 0 ≤ p ≤ 0.08, the spreading capabilities of the key nodes identified by the KEG algorithm are almost the same as those of the actual key nodes, indicating that it has higher precision in identifying the top 8% of the nodes compared with other methods. On the contrary, the K-shell decomposition algorithm generates the largest integral area, approximately 1.53 × 10 ⁻ ³, indicating the highest ranking error, which is closely related to the structural characteristics of the network.

Among them, Fig 4 (h), (i), and (j) are ablation experiments. That is, under the same conditions, the K-shell decomposition algorithm, information entropy, and Dijkstra algorithm are removed from the KEG algorithm, and the contribution of each module in the KEG algorithm is measured through the results. In the ablation experiment, the curve areas of the three graphs are smaller than those of the KEG algorithm (Fig 4 (a)), indicating that the accuracy is not as high as that of the KEG model. This result highlights the good robustness of the proposed integration strategy: the removal of any of the constituent methods will weaken the algorithm's ability to model the inherent complex patterns in the data. In summary, the results of the node spreading capabilities show that the KEG algorithm has higher precision than other methods.

#### 4.2.2 Verification of node importance by robustness analysis.

**Fig 5:**
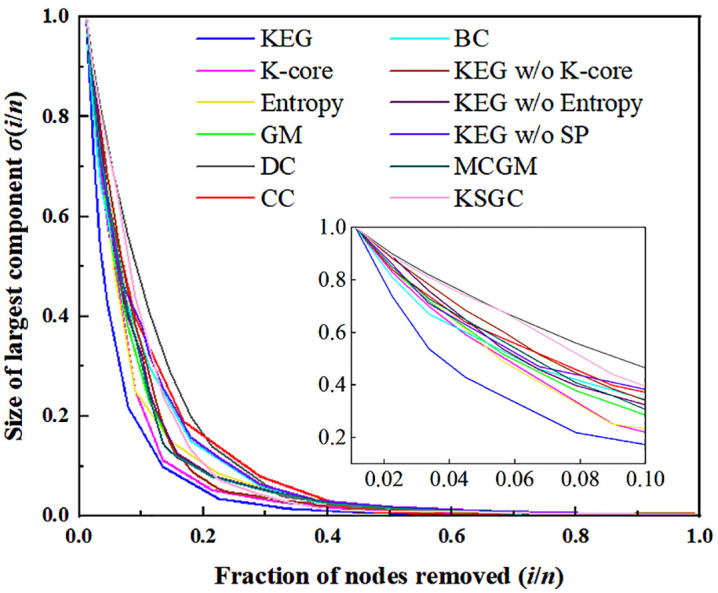
Robustness experiments of multiple algorithms in the network.

In robustness experiments, the impact of node removal on network structure and functionality is evaluated. Greater disruption implies higher criticality of the removed nodes [31]. Starting from a network with *n* nodes and *m* edges, nodes are ranked in descending order of importance and removed sequentially. Let σ (*i*/*n*) denote the relative size of the largest connected component after removing (*i*/*n*) nodes. A faster decline in σ (*i*/*n*) indicates higher accuracy of the ranking algorithm. As shown in Fig 5, σ (*i*/*n*) decreases with increasing (*i*/*n*), eventually approaching zero. The inset provides an enlarged view for the range 0 < *i*/*n* < 0.10.

The curve generated by the KEG algorithm decreases most rapidly as nodes are removed, signifying that its identified nodes occupy the most critical structural positions. As illustrated in the inset, betweenness centrality (BC) exhibits the second steepest descent in the removal ratio interval 0–0.04, since high-betweenness nodes often serve as bridges connecting network communities, rendering their removal highly disruptive. Thus, BC demonstrates relatively high accuracy in robustness assessment, second only to KEG. Notably, within the removal ratio range of 0.09–0.2, the K-shell model curve descends relatively fast. This occurs because the K-shell method prioritizes nodes in the core layer, which are densely interconnected; removal of such nodes initially has limited impact on connectivity. In contrast, nodes in intermediate cores play more crucial roles in maintaining global connectivity, and their removal causes greater fragmentation. Furthermore, degree centrality shows the largest errors in the interval 0–0.15, closeness centrality in 0.15–0.4, and betweenness centrality in 0.4–1, indicating their comparatively lower accuracy in identifying structurally vital nodes during progressive removal.

Fig 5 includes three curves derived from the ablation experiments, corresponding to the KEG w/o K-shell, KEG w/o entropy, and KEG w/o SP methods, respectively. These three curves exhibit a relatively fast declining rate, which indicates that the accuracy of these three ablation variants is higher than that of classical metrics and single algorithms, but lower than that of the original KEG algorithm. This observation demonstrates the effectiveness and robustness of the KEG algorithm.

#### 4.2.3 Verification of accident sensitivity.

From an accident-factor perspective, sensitivity analysis was performed to evaluate algorithm accuracy. The several methods were used to identify respective sets of key factors. By assuming the absence of each set, the reduction in accident frequency was measured to quantify the sensitivity of accidents to each factor group. A larger reduction indicates higher factor sensitivity, greater contribution to accidents, increased criticality, and higher algorithm accuracy.

**Fig 6:**
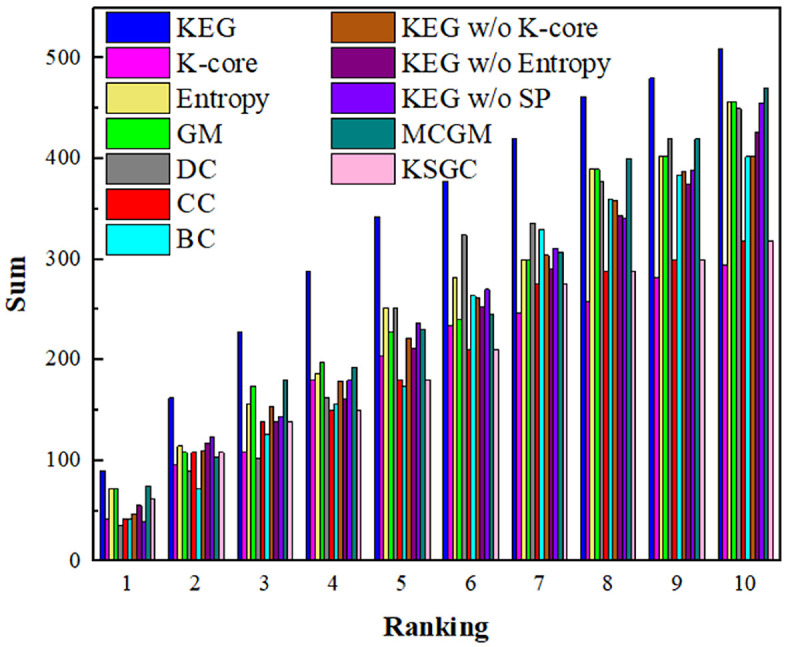
Total sum of accidents influenced by different key factors.

To facilitate comparison, the values in this row are visualized as a bar chart in Fig 6. The X-axis indicates the number of key factors, and the Y-axis represents the cumulative number of accidents. For instance, the dark blue bar corresponds to the KEG algorithm, showing that the top two key factors collectively account for 162 accidents. Compared to the other algorithms, the KEG algorithm yields the highest cumulative accident count, demonstrating its superior identification accuracy and underscoring the practical significance of this study.

Fig 6 includes results from the ablation experiments, corresponding to the KEG w/o K-shell, KEG w/o entropy, and KEG w/o SP methods, respectively. The column heights of these three methods are lower than those of the KEG algorithm. In addition, as comparative algorithms, the accuracy of the MCGM and KSGC algorithms is also lower than that of the KEG algorithm. This observation also demonstrates the effectiveness and robustness of the KEG algorithm.

### 4.3 Validation of robustness

To validate the robustness of the method, this section assesses its effectiveness and accuracy using an additional dataset sourced from the National Fire Incident Reporting System (NFIRS). The dataset comprises 1,204 building fire incidents recorded in Alaska from January 2012 to December 2024. Data preprocessing followed the same procedures applied to the California building fire dataset, resulting in 89 distinct factors categorized into 16 classes. A complete description of all variables and the classification scheme, consistent with that used for the Alaska dataset, is available in the supporting information (S1 File).

The basic attributes of the network are as follow: the number of nodes (𝑁) is 89, the number of edges (𝐸) is 1204, average degree (‹*k*›) is 27.1, assortative parameter (𝑟) is 0.33, propagation theory threshold (*α*_*c*_) is 0.07 and the actual transmission probability (*α*)is 0.12. To verify whether the constructed network conforms to the basic rules of complex networks, the scale-free and small-world characteristics of the accident network should be examined. The specific verification part can be found in the supporting information (S2 File).

The KEG algorithm is employed in this dataset to identify critical nodes and factors, while its effectiveness and precision are validated through Susceptible-Infected-Recovered (SIR) model simulations, network robustness analysis, and accident susceptibility analysis.

For the SIR-based evaluation, the precision function *ε*(*p*) is defined in Eq. (12) as $\varepsilon (p)=\text{1}-\frac{Mrank(p)}{Meff(p)}$, where *M*_*rank*_(*p*) denote the average propagation efficiency of the top *pN* nodes according to the model ranking and the true spreading-capability ranking, respectively. A smaller *ε*(*p*) indicates that the model's ranking better matches the actual influential nodes. To summarize the overall accuracy across all proportions of top-ranked nodes, we compute the area under the *ε*(*p*) curve over *p*∈[0,1]; a smaller area corresponds to higher overall precision. Table 1 reports these areas for each method, with the detailed calculation procedures described in Section 4.2.1.

**Table 1:** Comparison of area of precision functions for the influence ranking methods of nodes in the AK accident network (×10−5).

KEG	K-shell	Entropy	GM	DC	CC	BC	KEG w/o K-shell	KEG w/o entropy	KEG w/o SP	MCGM	KSGC
0.37	2.41	0.87	3.69	0.48	0.85	3.27	0.63	3.74	0.67	0.59	0.70

As shown in the Table 1, the area enclosed between the precision function of the KEG algorithm and the x-axis is the smallest, indicating that the KEG algorithm achieves the highest precision in identifying critical nodes.

**Fig 7:**
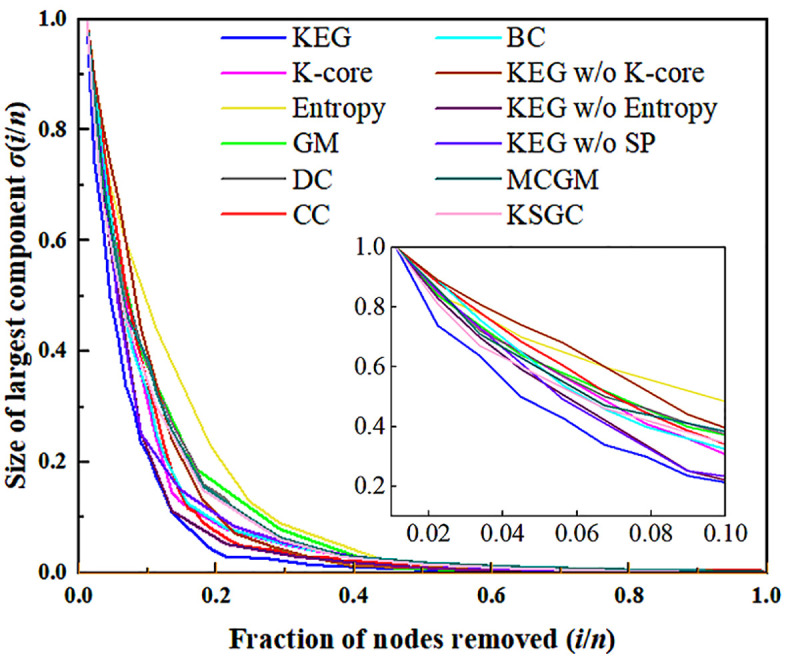
Robustness experiments of multiple algorithms.

Fig 7 shows the results of network robustness analysis. For the specific experimental steps, see 4.2.2. As shown in the Fig 7, the curve of the largest connected subset of the KEG algorithm drops the fastest, which proves that the attacked nodes with high rankings play an important role in the network, and also proves that the KEG algorithm has higher accuracy.

**Fig 8:**
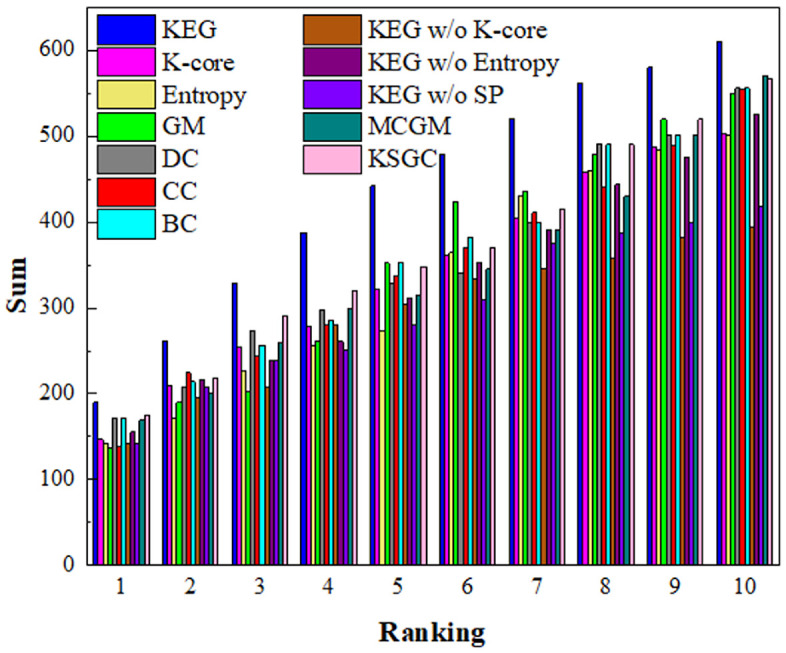
Total sum of accidents influenced by different key factors.

Fig 8 presents the experimental results of the accident susceptibility analysis, with detailed procedures provided in Section 4.2.3. As illustrated, under identical conditions of intervening with the same number of critical factors (i.e., preventing the occurrence of key factors), the bar representing the KEG algorithm attains the highest value. This indicates that incident outcomes are most sensitive to the critical factors identified by the KEG algorithm, thereby demonstrating its superior accuracy in identifying pivotal factors.

The accuracy comparison of the KEG algorithm with all other methods in the two sets of data can be found in the supporting information (S3 File).

## 5 Discussion

For building fire prevention, factor importance analysis serves as a crucial index for identifying key risk factors, providing a scientific basis for the development of effective prevention strategies. As shown in Table 1, the impact of different factors on building fires varies significantly. The KEG algorithm accurately outputs the important factors of building fires in California, factor 10 (Misuse of material or product, other) exerts the most pronounced influence. This factor encompasses a wide range of scenarios across the procurement, storage, and construction phases, accounting for 34% of all building fire causes, according to the National Fire Protection Association (2023). Moreover, the presence of vertical shaft structures in buildings can accelerate the vertical spread of fire, further amplifying the hazard associated with material misuse, making this factor a critical cause of fire incidents. Factor 6 (Multiple persons involved, includes gang activity) also ranks highly in importance. According to the Occupational Safety and Health Administration (OSHA), in 2024, large-scale commercial complexes increasingly involved cross-contracting and multi-party operations, with fire incidents in group work settings showing an annual growth rate of 12%. The third-ranked factor is Factor 73 (Outside/Open fire for debris or waste disposal). Data from the California Department of Forestry and Fire Protection (CAL FIRE) indicate that the annual compliance rate for waste-burning regulations is only 58%, with 41% of suburban building fires attributed to improper open burning. In addition, the probability of firebrand ignition in clusters of wooden structures reaches as high as 78%, according to the International Council on Monuments and Sites (ICOMOS). These findings highlight the critical importance of targeted fire prevention strategies based on empirical data and context-specific factor analysis.

A comprehensive analysis of the top 10 key factors identified by the KEG model reveals that they can be categorized into three main types: human-related factors, mechanical factors, and environmental factors, as summarized in Table 2. The Table 2 provides an interpretation and analysis of each key factor from two perspectives: driving causes of high frequency factors and transmission paths of strong correlation effects. This dual-perspective approach illustrates the underlying mechanisms by which these key factors contribute to the outbreak of building fires.

**Table 2:** The top ten key factors causing building fires.

Types of factors	Driving causes of high frequency factors	Transmission paths of strong correlation effects
Human factors	Behavior frequency· regulatory loopholes	Risk chain transmission (e.g., substance abuse → operational malpractice)
Mechanical factors	Equipment density· aging rate	Fault coupling (e.g., arc fault → ballast overheating)
Environmental factors	Space compression· climate aridity	Energy cascade (e.g., open burning → cross-structure ignition)

**Fig 9:**
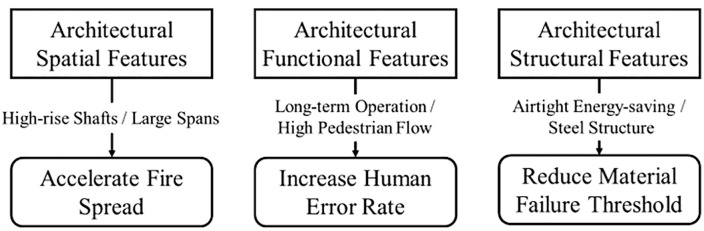
Three-dimensional impact model of building fire.

Fig 9 presents the three-dimensional impact model of building fires. The occurrence and progression of building fires are influenced by a range of architectural characteristics, including spatial layout, functional usage, and structural features. The associated risk factors derived from these characteristics can trigger fires or exacerbate their severity through specific mechanisms.

From the perspective of spatial layout, vertical shafts in high-rise buildings provide a rapid upward channel for hot air and flames, creating a chimney-like effect. As a result, heat sources present in such environments are more likely to cause large-scale fires, e.g., factors 11 (*Abandoned or discarded materials or products. Includes discarded cigarettes, cigars, tobacco embers, hot ashes, or other burning matter. Excludes outside fires left unattended.*), 12 (*Heat source too close to combustibles.), and 84 (Heat from direct flame, convection currents spreading from another fire.*). In terms of building functionality, prolonged work often means occupants remain in a working or active state for extended periods, which can lead to fatigue and reduced attention, e.g., factors 6 (*Multiple persons involved. Includes gang activity.*) and 2 (*Possibly impaired by alcohol or drugs. Includes people who fall asleep or act recklessly or carelessly as a result of drugs or alcohol. Excludes people who simply fall asleep.*), increasing the likelihood of human error, e.g., factor 10 (*Misuse of material or product, other.*). Simultaneously, continuous operation of equipment, e.g., factors 29 (*Electrical arcing.*) and 37 (*Fluorescent light ballast.*) raises the risk of mechanical failure. In high-footfall environments, the diversity of occupants and varying levels of familiarity with the premises make management more challenging. This can result in unsafe behaviors such as improper use of fire or electricity and blocked emergency exits. Collectively, these conditions significantly elevate the rate of human error—one of the leading causes of fires. For instance, factors such as 73 (*Outside/Open fire for debris or waste disposal.*) and 43 (*Installation deficiency.*) can directly trigger fire incidents. According to the perspective of building structural features, sealed energy-efficient designs are beneficial for energy conservation under normal conditions. However, during a fire, such designs can restrict airflow, causing heat and toxic gases to accumulate within the building and hindering timely ventilation. Elevated temperatures accelerate the aging and degradation of construction materials, weakening their fire resistance. In particular, steel structures pose significant risks—not only do they endanger the lives of occupants, but they also create substantial challenges for firefighting and rescue operations, increasing both the complexity and danger of emergency response.

## 6 Conclusion

This study introduces a novel complex network-based method for identifying key factors in building fires, offering substantial innovation, accuracy, and practical applicability. Unlike traditional approaches that rely on expert judgment or analytic hierarchy processes, the proposed technique constructs a complex network without prior knowledge, enabling objective and stable quantification of factor influences. By integrating K-shell, information entropy, and gravity models, the method significantly improves identification precision and offers a more effective tool for fire risk assessment.

The approach provides actionable insights for fire management and policy-making. Authorities can prioritize inspections and dynamic monitoring based on identified key factors, enabling efficient resource deployment and proactive interventions. Decision-makers can optimize emergency plans and prevention strategies, especially during high-risk periods, enhancing overall fire control effectiveness.

A limitation lies in its reliance on structured data, excluding unstructured sources like images or social media text. Future work will explore multimodal data fusion to further improve the comprehensiveness and robustness of fire risk assessment. In addition, the analysis of this paper does not consider the dynamic characteristics and severity of the accident. In subsequent research, relevant data will be collected with emphasis to make the analysis more comprehensive.

### Appendix. The symbol

**Table:** 

Symbol	The implications
*A*	The adjacency matrix
*a*_*ij*_	The element in the *i*th row and *j*th column in *A*
*D*	The set of degree for all nodes
*D*(*v*_*i*_)	The sum of the shortest paths
*d*_*i*_	The degree centrality of the *i*th node
*E*	The network edges set
*G*	The network of accidents
*G* ^*’*^	The sub-network of accidents
*H*	The information entropy
Δ*H*	The discrepancy between different information entropies
*Im*(*v*_*i*_)	The significance of node *v*_*i*_
*k*_*s*_	The core value
*M*_*rank*_(*p*)	The average propagation efficiency of the top *pN* nodes according to their influence
*M*_*eff*_(*p*)	The average propagation efficiency of the top *pN* nodes ranked according to their actual propagation capability
*N*	The node number in the network
*P*	The probability distribution
*p*	The proportion of evaluated nodes relative to the total network size
*r*	Assortative parameter
*S*_*ks*_	The set of factor nodes with degree being equal to *k*_*s*_
*V*	The network node set
*u*	The node
*W*	The edge weights set of network
*w*_*ij*_	The element in the *i*th row and *j*th column in *W*
*α*_*c*_	Propagation theory threshold
*ε*(*p*)	The accuracy of the model in identifying key nodes
σ (*i*/*n*)	The relative size of the largest connected component after removing (*i*/*n*) nodes

## Supporting information

S1 File Classification and code of accidents factors. (DOCX)

S2 File Scale-free and small-world properties of the accident network. (DOCX)

S3 File Comparison of the KEG against other methods across all validation metrics. (DOCX)

## References

[1] Zhao E, Wang N, Cui S, Zhao R, Yu Y. Identification method of forest fire risk factors and their coupling relationship driven by attribute dependence. Int J Disaster Risk Red. 2025;125:105529. doi: 10.1016/j.ijdrr.2025.105529

[2] Kim J, Shan Y, Kim S, Song D, Park H, Bang C. Factors influencing fire safety on building construction sites: a fire officer’s perspective. J Constr Eng Manage. 2021;147(10). doi: 10.1061/(asce)co.1943-7862.0002144

[3] Kim J-S, Kim B-S. Analysis of fire-accident factors using big-data analysis method for construction areas. KSCE J Civil Eng. 2018;22(5):1535–43. doi: 10.1007/s12205-017-0767-7

[4] Cai QJ, Zeng AC, Su ZW, Guo FT. Driving factors of forest fire in Zhejiang province based on logistic regression model. J Northwest A & F Univ (Natural Science Edition). 2020;48(02):102–9.

[5] Aamodt E. Analysis of a large fire in an apartment building used for social housing in Norway, Safety Science. 185, 000 (2025).

[6] Kim MO, Kim K, Yun JH, Kim MK. Fire risk assessment of cable bridges for installation of firefighting facilities. Fire Safety J. 2020;115:103146. doi: 10.1016/j.firesaf.2020.103146

[7] Zhang J, Huang L, Chen T, Su G. Simulation based analysis of electrical fire risks caused by poor electric contact between plug and receptacle. Fire Safety J. 2021;126:103434. doi: 10.1016/j.firesaf.2021.103434

[8] Tang S. Epidemiology of dwelling fires in England 2010–2023. Fire Safety J. 151(2025).

[9] Aydin M, Akyuz E, Boustras G. A holistic safety assessment for cargo holds and decks fire & explosion risks under fuzzy Bayesian network approach. Safety Science. 2024;176:106555. doi: 10.1016/j.ssci.2024.106555

[10] Vermina Plathner F, Sjöström J, Granström A. Garden structure is critical for building survival in northern forest fires – An analysis using large Swedish wildfires. Safety Science. 2023;157:105928. doi: 10.1016/j.ssci.2022.105928

[11] Briglia G, Immovilli F, Cocconcelli M, Lippi M. Bearing Fault Detection and Recognition From Supply Currents With Decision Trees. IEEE Access. 2024;12:12760–70. doi: 10.1109/access.2023.3348245

[12] Katicha SW, Flintsch GW. A kernel density empirical Bayes (KDEB) approach to estimate accident risk. Accid Anal Prev. 2023;186:107039. doi: 10.1016/j.aap.2023.107039 36989959

[13] Yang Y, Chen G, Reniers G. Vulnerability assessment of atmospheric storage tanks to floods based on logistic regression. Reliability Eng Syst Safety. 2020;196:106721. doi: 10.1016/j.ress.2019.106721

[14] Zhou J, Xu W, Guo X, Ding J. A method for modeling and analysis of directed weighted accident causation network (DWACN). Physica A: statistical mechanics and its applications. 2015;437:263–77. doi: 10.1016/j.physa.2015.05.112

[15] Ma X, Deng W, Qiao W, Luo H. A novel methodology concentrating on risk propagation to conduct a risk analysis based on a directed complex network. Risk Anal. 2022;42(12):2800–22. doi: 10.1111/risa.13870 35028963

[16] Chen H, Zhang L, Ran L. Vulnerability modeling and assessment in urban transit systems considering disaster chains: a weighted complex network approach. Int J Disaster Risk Red. 2021;54:102033. doi: 10.1016/j.ijdrr.2020.102033

[17] Hu LW, Fan ZJ, Zhang SH, Guo Z, Yin XF. Risk propagation mechanism and application of urban traffic congestion factors based on complex networks. J Transp Syst Eng Inform Tech. 2021;21(02):224–30.

[18] Guo JL. Complex networks and dynamic evolution models of human behavior. Science Press; 2013.

[19] Zhen L, Zhang Y, Li Z. Graph constraints refined for transitive relations. Knowledge-Based Syst. 2023;268:110458. doi: 10.1016/j.knosys.2023.110458

[20] Moreno Y, Nekovee M, Pacheco AF. Dynamics of rumor spreading in complex networks. Phys Rev E Stat Nonlin Soft Matter Phys. 2004;69(6 Pt 2):066130. doi: 10.1103/PhysRevE.69.066130 15244690

[21] Krackhardt D. Assessing the political landscape: structure, cognition, and power in organizations. Administ Science Quart. 1990;35(2):342. doi: 10.2307/2393394

[22] Freeman LC. A set of measures of centrality based on betweenness. Sociometry. 1977;40(1):35. doi: 10.2307/3033543

[23] Nandi S, Malta MC, Maji G, Dutta A. IC-SNI: measuring nodes’ influential capability in complex networks through structural and neighboring information. Springer Nature Link. 67, 1309–50 (2025).

[24] Nandi S, Dutta A. Local closeness gravity model to identify the vital nodes in complex networks. In: 2024 16th International Conference on COMmunication Systems & NETworkS (COMSNETS), 2024. 864–72. doi: 10.1109/comsnets59351.2024.10426978

[25] Li Z, Huang X. Identifying influential spreaders by gravity model considering multi-characteristics of nodes. Sci Rep. 2022;12(1):9879. doi: 10.1038/s41598-022-14005-3 35701528 PMC9197977

[26] Yang X, Xiao F. An improved gravity model to identify influential nodes in complex networks based on k-shell method. Knowledge-Based Systems. 2021;227:107198. doi: 10.1016/j.knosys.2021.107198

[27] Cox W. California: Most Urban and Densest Urban State; 2023. https://www.newgeography.com/content/007707-california-most-urban-and-densest-urban-state

[28] Steinberg I. Las Vegas building destroyed in blaze had no fire sprinklers, county says; 2025. https://preview.reviewjournal.com/local/local-las-vegas/las-vegas-building-destroyed-in-fire-had-no-fire-sprinklers-county-says-3112638/

[29] U.S. Fire Administration: Working for a fire safety America. https://www.usfa.fema.gov/nfirs/

[30] Kitsak M, Gallos LK, Havlin S, Liljeros F, Muchnik L, Stanley HE, et al. Identification of influential spreaders in complex networks. Nature Phys. 2010;6(11):888–93. doi: 10.1038/nphys1746

[31] Zhao N, Bao J, Chen N. Ranking influential nodes in complex networks with information entropy method. Complexity. 2020;2020:1–15. doi: 10.1155/2020/5903798

[32] Li Z, Ren T, Ma X, Liu S, Zhang Y, Zhou T. Identifying influential spreaders by gravity model. Sci Rep. 2019;9(1):8387. doi: 10.1038/s41598-019-44930-9 31182773 PMC6557850

[33] Ren XL, Lv LY. Review of ranking nodes in complex networks. Chinese Science Bulletin. 2014;59(13):1175–97.

