# Supplementary material for: Identifying key factors in building fires: A novel approach fusing K-shell entropy gravity
Source: PLoS One. 2026 Jun 5;21(6):e0350804. doi: 10.1371/journal.pone.0350804 (PMC13240863; doi:10.1371/journal.pone.0350804)
Supplement: S1 File — (DOCX) [file pone.0350804.s001.docx]

# Classification and Code of Accidents Factors

Based on the above data sources and processing methods, the specific classification and numbering of accidents factors are presented in Table 1.

Table 1 The specific classification and numbering of accidents factors

| **Categories** | **Codes** | **Factors Contributing** |
| --- | --- | --- |
| **Misuse of Material or Product** | 11 | Abandoned or discarded materials or products. Includes discarded cigarettes, cigars, tobacco embers, hot ashes, or other burning matter. Excludes outside fires left unattended. |
|  | 12 | Heat source too close to combustibles. |
|  | 13 | Cutting, welding too close to combustibles. |
|  | 14 | Flammable liquid or gas spilled. Excludes improper fueling technique and release due to improper container. |
|  | 15 | Improper fueling technique. Includes overfueling, failure to ground. Excludes fuel spills and using the improper fuel. |
|  | 16 | Flammable liquid used to kindle fire. |
|  | 18 | Improper container or storage procedure. Includes gasoline in unimproved containers, gas containers stored at excessive temperature, and storage conditions that lead to spontaneous ignition. |
|  | 19 | Playing with heat source. Includes playing with matches, candles, and lighters and bringing combustibles into a heat source. |
|  | 10 | Misuse of material or product, other. |
| **Mechanical Failure, Malfunction** | 21 | Automatic control failure. |
|  | 22 | Manual control failure. |
|  | 23 | Leak or break. Includes leaks or breaks of containers or pipes. Excludes operational deficiencies and spill mishaps. |
|  | 25 | Worn out. |
|  | 26 | Backfire. Excludes fires originating as a result of hot catalytic converters. |
|  | 27 | Improper fuel used. Includes the use of gasoline in a kerosene heater and the like. |
|  | 20 | Mechanical failure, malfunction, other. |
| **Electrical Failure, Malfunction** | 31 | Water-caused short-circuit arc. |
|  | 32 | Short-circuit arc from mechanical damage. |
|  | 33 | Short-circuit arc from defective, worn insulation. |
|  | 34 | Unspecified short-circuit arc. |
|  | 35 | Arc from faulty contact, broken conductor. Includes broken power lines and loose connections. |
|  | 36 | Arc, spark from operating equipment, switch, or electric fence. |
|  | 37 | Fluorescent light ballast. |
|  | 30 | Electrical failure, malfunction, other. |
| **Design, Manufacturing, Installation Deficiency** | 41 | Design deficiency. |
|  | 42 | Construction deficiency. |
|  | 43 | Installation deficiency. |
|  | 44 | Manufacturing deficiency. |
|  | 40 | Design, manufacturing, installation deficiency, other. |
| **Operational Deficiency** | 51 | Collision, knock down, run over, turn over. Includes automobiles and other vehicles. |
|  | 52 | Accidentally turned on, not turned off. |
|  | 53 | Equipment unattended. |
|  | 54 | Equipment overloaded. |
|  | 55 | Failure to clean. Includes lint and grease buildups in chimneys, stove pipes. |
|  | 56 | Improper startup/shutdown procedure. |
|  | 57 | Equipment not used for purpose intended. Excludes overloaded equipment. |
|  | 58 | Equipment not operated properly. |
|  | 50 | Operational deficiency, other. |
| **Natural Condition** | 61 | High wind. |
|  | 62 | Storm. |
|  | 63 | High water, including floods. |
|  | 64 | Earthquake. |
|  | 66 | Animal. |
|  | 60 | Natural condition, other. |
| **Fire Spread** | 72 | Rekindle. |
|  | 73 | Outside/Open fire for debris or waste disposal. |
|  | 74 | Outside/Open fire for warming or cooking. |
|  | 70 | Fire spread or control, other. |
| **Human Factors** | 1 | Asleep. Includes fires that result from a person falling asleep while smoking. |
|  | 2 | Possibly impaired by alcohol or drugs. Includes people who fall asleep or act recklessly or carelessly as a result of drugs or alcohol. Excludes people who simply fall asleep. |
|  | 3 | Unattended or unsupervised person. Includes “latch key” situations whether the person involved is young or old and situations where the person involved lacked supervision or care. |
|  | 4 | Possibly mentally disabled. Excludes impairments of a temporary nature such as those caused by drugs or alcohol. |
|  | 5 | Physically disabled. |
|  | 6 | Multiple persons involved. Includes gang activity. |
|  | 7 | Age was a factor. |
| **Fire Extinguishing System** | 8 | System operated and was not effective. |
|  | 9 | System did not operate. |
|  | 0 | No sprinkler system installed. |
| **Operating Equipment as a Heat Source** | 17 | Heat from operating equipment, other. |
|  | 24 | Spark, ember, or flame from operating equipment. |
|  | 28 | Radiated or conducted heat from operating equipment. |
|  | 29 | Electrical arcing. |
| **Hot or Smoldering Object as a Heat Source** | 38 | Hot or smoldering object, other. |
|  | 39 | Heat, spark from friction. Includes overheated tires. |
|  | 45 | Molten, hot material. Includes molten metal, hot forging, hot glass, hot metal fragment, brake shoe, hot box, and slag from arc welding operations. |
|  | 46 | Hot ember or ash. Includes hot coals, coke, and charcoal; and sparks or embers from a chimney that ignite the roof of the same structure. Excludes flying brand, embers, and sparks; and embers accidentally escaping from operating equipment. |
| **Explosives, Fireworks as a Heat Source** | 47 | Munitions. Includes bombs, ammunition, and military rockets. |
|  | 48 | Fireworks. Includes sparklers, paper caps, party poppers, and firecrackers. |
|  | 49 | Incendiary device. Includes Molotov cocktails and arson sets. |
| **Other Open Flame or Smoking Materials as a Heat Source** | 59 | Heat from open flame or smoking materials, other. |
|  | 65 | Cigarette. |
|  | 67 | Pipe or cigar. |
|  | 68 | Heat from undetermined smoking material. |
|  | 69 | Match. |
|  | 71 | Lighter: cigarette lighter, cigar lighter. |
|  | 75 | Candle. |
|  | 76 | Warning or road flare; fusee. |
|  | 77 | Flame/torch used for lighting. Includes gas light and gas-/liquid-fueled lantern. |
| **Chemical, Natural Heat Sources as a Heat Source** | 78 | Chemical, natural heat sources, other. |
|  | 79 | Sunlight. Usually magnified through glass, bottles, etc. |
|  | 80 | Spontaneous combustion, chemical reaction. |
|  | 81 | Lightning discharge. |
|  | 82 | Other static discharge. Excludes electrical arcs or sparks. |
| **Heat Spread From Another Fire as a Heat Source** | 83 | Heat spread from another fire, other. |
|  | 84 | Heat from direct flame, convection currents spreading from another fire. |
|  | 85 | Radiated heat from another fire. Excludes heat from exhaust systems of fuel-fired, fuel-powered equipment. |
|  | 86 | Flying brand, ember, spark. Excludes embers, sparks from a chimney igniting the roof of the same structure. |
|  | 87 | Conducted heat from another fire. |
| **Other Heat Sources** | 88 | Multiple heat sources, including multiple ignitions. If one type of heat source was primarily involved, use that classification. |
