# Supplementary material for: Identifying key factors in building fires: A novel approach fusing K-shell entropy gravity
Source: PLoS One. 2026 Jun 5;21(6):e0350804. doi: 10.1371/journal.pone.0350804 (PMC13240863; doi:10.1371/journal.pone.0350804)
Supplement: S2 File — (DOCX) [file pone.0350804.s002.docx]

# Scale-free and small-world properties of the accident network

## (1) California State

To verify whether the constructed network conforms to the basic rules of complex networks, the scale-free and small-world characteristics of the accident network should be examined. A scale-free network is characterized by a degree distribution that follows a power-law decay. In such networks, node connectivity is highly uneven: a few nodes have many connections, while most have only a few. The degree distribution, which represents the probability of node degrees across the network, plays a key role in defining the scale-free property. Due to the relatively small size of the accident network and its heavy-tailed degree distribution, it is recommended to measure the cumulative degree distribution. Similar to the degree distribution, the cumulative degree distribution represents the fraction of nodes with a degree more than or equal to *k*. The cumulative degree distribution of the California State accident network is shown in Figure 1. From this graph, it can be clearly observed that the degree distribution *p*(*k*) exhibits an approximate power-law decay with $p\left( k \right) \sim0.932k^{-0.398}(R^{2}=0.825)$, $\gamma_{cum}=0.398$. Therefore, the degree distribution exponent $\gamma=\gamma_{cum}+1$ is approximately 2, indicating that this network possesses the characteristics of a scale-free network.


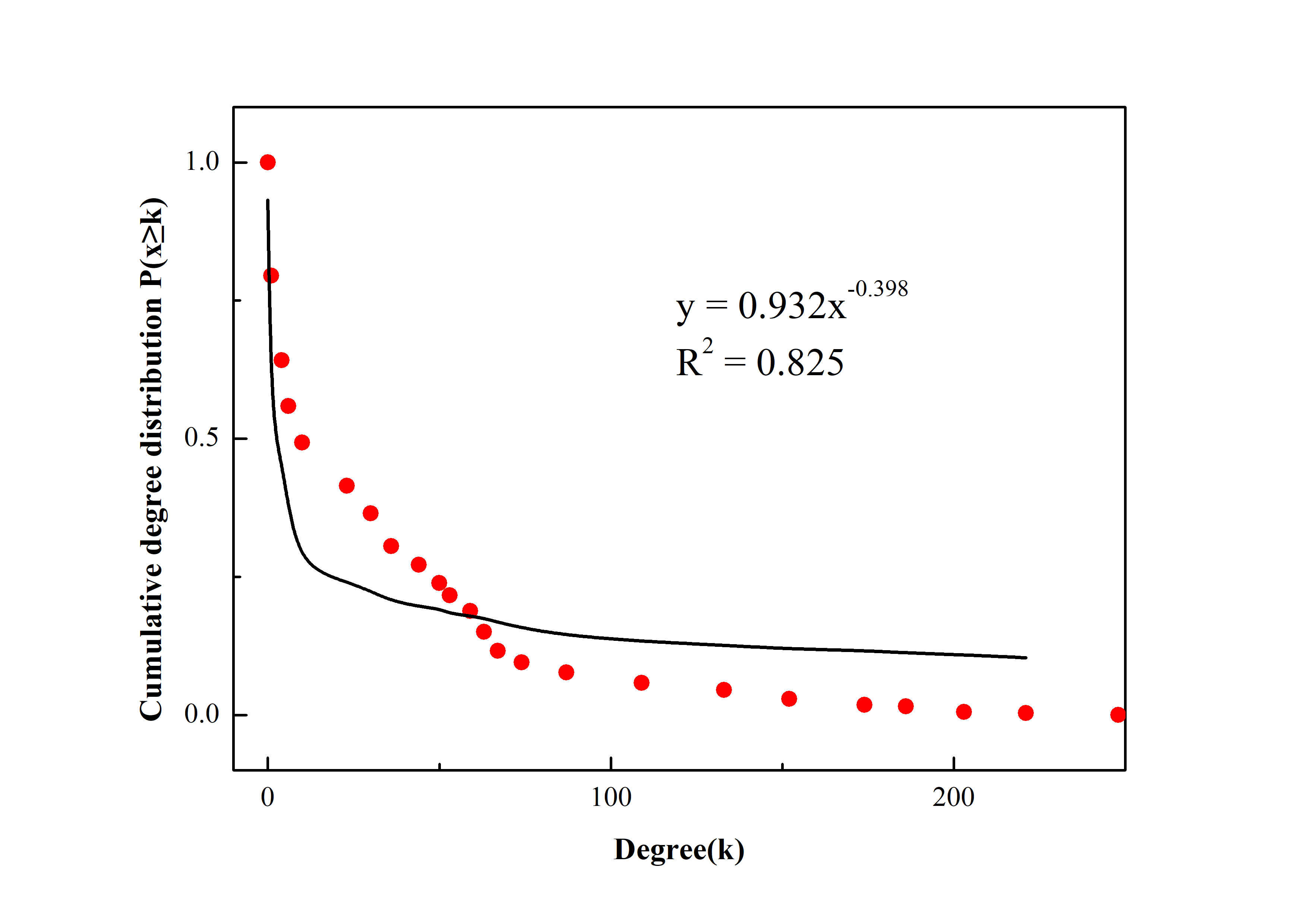


Fig. 1. The cumulative degree distribution of the California State accident network

In addition to the scale-free property, another characteristic observed in various real networks is the small-world property. According to this, most nodes are not directly connected, but they can be connected by one another through a small number of steps. Small-world networks usually have smaller average path lengths and higher values of clustering coefficients. These two features serve as the basis for examining whether the accident network satisfies the small-world characteristics. In a network, the distance between any two nodes is defined as the minimum number of edges required to connect them, and the average path length is a global property that represents the average number of edges required to reach any two nodes. The mathematical expression is shown in formula 1:

| $L=\frac{1}{N(N-1)}\sum_{i,j\in N,i\neq j} d_{ij}$ | (1) |
| --- | --- |

where *N* represents the number of nodes in the network, and $d_{ij}$ represents the shortest path length between nodes *i* and *j*. By calculating the formula, the average path length of the accident network is determined to be 2.295.

As shown in formula 2, ${CC}_{i}$ is the clustering coefficient of node *i*, $E_{i}$ is the number of edges of node *i*, $K_{i}$ is degree, representing that node *i* connected with $K_{i}$ nodes. Thus, the clustering coefficient of the network is 0.371.

| ${CC}_{i}=\frac{2E_{i}}{K_{i}(K_{i}-1)}$ | (2) |
| --- | --- |

To clarify, 100 scale-free networks of similar size to the accident network are randomly generated. According to formula s 1 and 2, the mean of the average path length of these networks is 2.706, while the mean clustering coefficient is 0.093. The average path length of the accident network is found to be smaller than that of the randomly generated scale-free networks, and the clustering coefficient of the accident network is significantly higher than that of the randomly generated scale-free networks. The presence of small-world phenomena in the accident network indicates close interrelations among the accidents. Therefore, it is necessary to identify the key nodes and select the key factors.

## (2) Alaska State

Similar to California, the cumulative degree distribution of the Alaska State accident network is shown in Figure 2. From this graph, it can be clearly observed that the degree distribution *p*(*k*) exhibits an approximate power-law decay with $p\left( k \right) \sim3.838k^{-0.928}(R^{2}=0.7317)$, $\gamma_{cum}=0.928$. Therefore, the degree distribution exponent $\gamma=\gamma_{cum}+1$ is approximately 2, indicating that this network possesses the characteristics of a scale-free network.


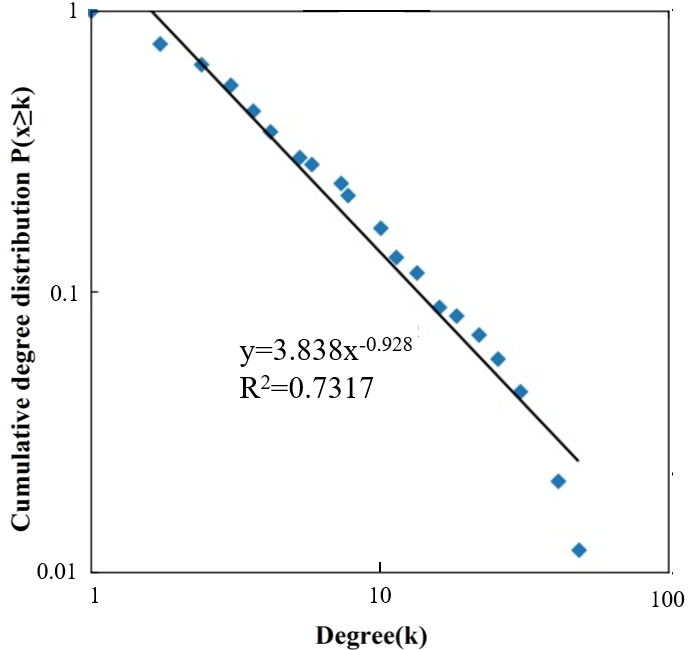


Fig. 2. The cumulative degree distribution of the Alaska State accident network

For the small - world property, by calculating the formula (1), the average path length of the accident network is determined to be 2.363. The clustering coefficient of the network is 0.428 based on formula (2). 100 scale-free networks of similar size to the accident network are randomly generated. According to formula (1) and (2), the mean of the average path length of these networks is 3.529, while the mean clustering coefficient is 0.104. The average path length of the accident network is found to be smaller than that of the randomly generated scale-free networks, and the clustering coefficient of the accident network is significantly higher than that of the randomly generated scale-free networks. The presence of small-world phenomena in the network indicates close interrelations among the accidents. Therefore, this network conforms to the laws of complex networks and relevant algorithms can be applied.
