# Supplementary material for: Identifying key factors in building fires: A novel approach fusing K-shell entropy gravity
Source: PLoS One. 2026 Jun 5;21(6):e0350804. doi: 10.1371/journal.pone.0350804 (PMC13240863; doi:10.1371/journal.pone.0350804)
Supplement: S3 File — (DOCX) [file pone.0350804.s003.docx]

# Comparison of the KEG against other methods across all validation metrics

The performance comparison results of the KEG algorithm versus other methods across all validation metrics in two datasets, including the SIR experiment, robustness analysis experiment, and accident sensitivity analysis experiment. Specifically, the "Propagation capacity" column represents the precise function value of the propagation capacity of identified important nodes in the SIR experiment. A smaller value in this column indicates more accurate identification of important nodes. As observed from the table, in both datasets, the KEG algorithm exhibits the smallest value in this column and thus the highest accuracy compared to other methods.

The "Robustness" column denotes the relative size of the largest component of the remaining network, calculated after attacking nodes ranked among the top 5%, 10%, 15%, and 20% in terms of importance during the network robustness analysis experiment. The table reveals that, in both datasets, the KEG algorithm results in the smallest largest component relative to other methods, demonstrating that the attacked nodes are those with greater influence in the network. The "Sensitivity" column represents the change in the number of accidents, calculated after removing the top 5 and top 10 factors ranked by importance in the sensitivity analysis experiment. It can be seen from the table that removing the important factors identified by the KEG algorithm leads to the most significant impact on the number of accidents. Additionally, both datasets confirm that the KEG algorithm achieves high accuracy, indicating that this algorithm possesses a certain degree of robustness.

This table not only compares the accuracy of important factors identified by the KEG algorithm with those identified by K-shell decomposition algorithm, information entropy, gravity model, degree centrality, closeness centrality, and betweenness centrality, but also includes the results of ablation analysis. Among them, the KEG w/o K-shell, KEG w/o entropy, and KEG w/o SP methods refer to the three comparative variants of the KEG algorithm, which are obtained by removing the K-shell decomposition algorithm, information entropy, and shortest path (SP) components from the original KEG algorithm, respectively. As can be observed from the table, in all three experiments, the accuracy of these three comparative variants is significantly lower than that of the original KEG algorithm, which verifies that the KEG algorithm has high effectiveness and accuracy.

In conclusion, the KEG algorithm possesses both high robustness and accuracy, enabling it to identify important factors and thereby achieve the goal of effectively managing and controlling building fires.

Table 1 Comparison of the experimental performance of the KEG algorithm and other methods in two groups of data

| **Method** | **Propagation capacity**  **(×10^-5^)** | **Robustness** | | | | **Sensitivity** | |
| --- | --- | --- | --- | --- | --- | --- | --- |
|  |  | **5%** | **10%** | **15%** | **20%** | **5** | **10** |
| **KEG** | **10.90** | **0.43** | **0.17** | **0.09** | **0.05** | **342** | **510** |
| **K-shell** | 153.00 | 0.59 | 0.22 | 0.10 | 0.06 | 204 | 294 |
| **Entropy** | 14.10 | 0.61 | 0.23 | 0.15 | 0.10 | 252 | 456 |
| **GM** | 92.30 | 0.62 | 0.28 | 0.15 | 0.09 | 228 | 456 |
| **DC** | 14.20 | 0.75 | 0.46 | 0.29 | 0.16 | 252 | 450 |
| **CC** | 11.20 | 0.64 | 0.37 | 0.25 | 0.16 | 180 | 318 |
| **BC** | 47.40 | 0.60 | 0.34 | 0.23 | 0.13 | 174 | 402 |
| **KEG w/o K-shell** | 14.80 | 0.68 | 0.34 | 0.15 | 0.07 | 222 | 403 |
| **KEG w/o entropy** | 16.80 | 0.65 | 0.32 | 0.15 | 0.09 | 211 | 426 |
| **KEG w/o SP** | 19.50 | 0.63 | 0.38 | 0.23 | 0.14 | 237 | 455 |
| **KEG** | **0.37** | **0.50** | **0.21** | **0.10** | **0.04** | **442** | **610** |
| **K-shell** | 2.41 | 0.65 | 0.30 | 0.13 | 0.09 | 322 | 503 |
| **Entropy** | 0.87 | 0.70 | 0.48 | 0.35 | 0.21 | 274 | 502 |
| **GM** | 3.69 | 0.64 | 0.37 | 0.25 | 0.16 | 352 | 550 |
| **DC** | 0.48 | 0.65 | 0.37 | 0.25 | 0.14 | 328 | 556 |
| **CC** | 0.85 | 0.68 | 0.34 | 0.15 | 0.07 | 337 | 555 |
| **BC** | 3.27 | 0.65 | 0.32 | 0.15 | 0.09 | 352 | 556 |
| **KEG w/o K-shell** | 0.63 | 0.74 | 0.39 | 0.21 | 0.10 | 304 | 394 |
| **KEG w/o entropy** | 3.74 | 0.59 | 0.22 | 0.10 | 0.06 | 311 | 526 |
| **KEG w/o SP** | 0.67 | 0.61 | 0.23 | 0.15 | 0.10 | 280 | 418 |
